# Corticosteroid-induced manic and/or psychotic symptoms: a systematic review

**DOI:** 10.3389/fphar.2025.1628765

**Published:** 2025-07-22

**Authors:** Sara Gostoli, Danilo Carrozzino, Giulia Raimondi, Regina Subach, Graziano Gigante, Chiara Rafanelli

**Affiliations:** ^1^ Department of Psychology “Renzo Canestrari”, University of Bologna, Bologna, Italy; ^2^ Department of Developmental and Social Psychology, Sapienza University of Rome, Rome, Italy

**Keywords:** adverse reactions, clinimetrics, corticosteroid-induced psychiatric symptoms, mania, psychosis

## Abstract

**Introduction:**

Given their immunosuppressive and anti-inflammatory properties, corticosteroids are widely used. However, corticosteroid-induced psychiatric effects are deeply concerning, since they can be severe and require accurate differential diagnosis from primary psychiatric disorders. Among corticosteroid-induced psychiatric symptoms, what kind of patients under corticosteroids develop manic and/or psychotic symptoms and which risk factors are associated with their development remain unclear.

**Methods:**

The present systematic review aimed at giving a comprehensive overview of corticosteroids-induced symptoms of mania and/or psychosis and at examining the clinical factors that might increase the risk of developing these adverse reactions. According to PRISMA guidelines, this systematic review included 40 papers (clinical cases = 34; quantitative research = 6).

**Results:**

In 64.7% of clinical cases and 33.3% of research studies reviewed, patients taking corticosteroids presented with both manic and psychotic symptoms; in 11.8% of clinical cases and 1 (out of 6) quantitative research patients under corticosteroids presented with manic symptoms only, whereas in 23.5% of clinical cases and 3 quantitative studies, psychotic symptoms only. Prolonged and high-dose corticosteroid therapy, pre-existing psychiatric conditions, older age and female sex represent risk factors, which are likely to increase individual susceptibility to corticosteroid-induced symptoms of mania and/or psychosis.

**Discussion:**

Although manic and psychotic symptoms often coexist in patients taking corticosteroids, the direction and nature of this relationship (e.g., which symptoms appear first, their interaction and progression over time) remain unclear. Clinicians prescribing corticosteroids might take advantage of clinimetric methods, which may allow a substantial improvement in the early detection and evaluation of severity of corticosteroid-induced manic and/or psychotic symptoms.

## 1 Introduction

Corticosteroids, also known as steroids, are one of the most widely used drugs in modern medicine given their immunosuppressive properties and anti-inflammatory effects. The most frequently prescribed are prednisone, methylprednisolone, cortisone and hydrocortisone (Dabbah-Assadi et al., 2022). The term “steroids,” which includes glucocorticoids and mineralocorticoids, encompasses substances such as cortisone, cholesterol, and other hormones with similar chemical structures (Thibaut, 2019). Endogenous steroids, such as cortisol and hydrocortisone, are naturally produced by the body in the adrenal glands, and help regulating metabolism, inflammation, and maintain homeostasis, specifically during exposure to stressors (Rhen and Cidlowski, 2005; Breen et al., 2019).

Glucocorticoids have several endocrinological properties, influencing glucose and lipid metabolism, bone and cartilage health, protein metabolism, muscle function, gastric secretion, cardiovascular system, hematopoietic tissue, and reproductive physiology (Ramamoorthy and Cidlowski, 2016). They also affect appetite, sleep-wake cycles, and cognitive functions such as learning and memory through interactions with receptors in the prefrontal cortex, hippocampus, and amygdala (Frau et al., 2020).

Pharmacological therapy with corticosteroids has been used to treat patients with various inflammatory conditions, including intestinal, allergic, and immunological disorders (Williams, 2018; Reichardt et al., 2021). In addition to their therapeutic or beneficial effects, the use of corticosteroids (i.e., either short-term or prolonged) can lead to significant side effects, both physical and psychiatric (Dabbah-Assadi et al., 2022). For example, from a physical perspective, short-term corticosteroid use can cause skin problems (e.g., acne, bruising, hyperpigmentation, atopic dermatitis) (Drucker et al., 2018), whilst long-term use is associated with musculoskeletal conditions (e.g., osteoporosis), myopathies, endocrine and metabolic syndromes, cardiovascular conditions, gastrointestinal disorders, dermatological diseases, ophthalmologic issues and immunologic problems (Oray et al., 2016).

### 1.1 Psychiatric symptoms

Research on corticosteroid-induced psychiatric effects began over 60 years ago (Clark et al., 1953; Ritchie, 1956). Early studies highlighted a wide range of psychiatric symptomatology, including affective, psychotic symptoms, and cognitive dysfunctions, sometimes manifesting as reversible dementia (Ling, 1981; Brown et al., 1999). As Thibaut (2019) noted in her editorial, a weighted average of 6% of severe depression, mania, and psychosis was found, whereas 28% of patients taking corticosteroids exhibited mild to moderate psychiatric symptoms (Lewis and Smith, 1983).

Affective symptoms are one of the most common psychiatric side effects associated with corticosteroids treatment (Manzo et al., 2018). Specifically, short-term and high doses, particularly above 40 mg of prednisone per day, are more likely to trigger such symptoms. Studies have also showed an association between corticosteroid use and depressive and hypomania symptoms, especially in long-term therapy, which can also lead to suicidal behaviors in depressed patients (Morrow et al., 2015).

Delirium can also arise from corticosteroid use (Ishikawa et al., 2018). This condition often involves sensory overstimulation and attentional deficits, exacerbating other delirium etiologies. Delirium in corticosteroid users can manifest as paranoia, confusion, and severe disorientation, potentially leading to significant distress and functional impairment in affected individuals.

Findings of a recent review showed that glucocorticoid users reported significantly higher scores on depression and mania rating scales compared to nonusers (Koning et al., 2024). Prevalence rates indicated a pooled proportion of 22% for symptoms of depression, 11% for symptoms of mania, 8% for symptoms of anxiety, 16% for delirium, and 52% for behavioral changes among glucocorticoid users (Koning et al., 2024).

In another recent systematic review on the association between the use of corticosteroids and mania (De Bock and Sienaert, 2024), even higher prevalence rates have been found, with steroid-induced mania which was reported in 32 (84.2%) and hypomania in 6 (15.8%) of the 38 case studies under evaluation. However, as the authors of this systematic review noted, the absence of standardized diagnostic criteria for corticosteroid-induced mania was a significant source of bias, which may lead to potential misclassification and variability in reported prevalence rates (De Bock and Sienaert, 2024). The existing literature on this topic (Gable and Depry, 2015; Jasani et al., 2021; Sofía-Avendaño-Lopez et al., 2024) also suggested that compared to individuals with primary mania, patients with steroid-induced mania are more likely to develop other psychotic symptoms, which are not always strictly congruent to mania. It remains, however, unclear what kind of patients under corticosteroids actually develop manic and/or psychotic symptoms. The same controversial issue applies to risk factors for the development of manic and/or psychotic symptoms. Indeed, despite the number of studies on this topic, findings remain inconclusive. This implies the need for an updated systematic review of the literature to provide a more comprehensive clinical picture regarding symptoms of mania and/or psychosis in patients taking these medications. Although several recent reviews have been conducted on corticosteroid-induced psychiatric adverse effects (Alturaymi et al., 2023; De Bock and Sienaert, 2024; Koninget al., 2024), the present review includes studies that were not cited in the previous ones, thereby enriching the field with new evidence. Given that these psychiatric side-effects are often subtle and come to the attention of the clinician only when they become severe, our review will also provide methodological recommendations and strategies for the early detection of corticosteroid-induced psychiatric symptoms.

Based on these premises, this is the first systematic review of studies aimed at a comprehensive assessment of symptoms of mania and/or psychosis in patients taking corticosteroids. The major aim was not only to understand clinical trajectories and distinctive manifestations of corticosteroid-induced mania and/or psychosis but also to examine the clinical factors or moderators that might increase the risk of developing these adverse reactions following the administration of corticosteroids.

## 2 Methods

The present study employs a systematic literature review research design.

### 2.1 Search strategy

The systematic review was conducted in accordance with the Preferred Reporting Items for Systematic Review and Meta-Analysis (PRISMA) guidelines (Moher et al., 2009; Page et al., 2021). The systematic search of the literature from 2013 up to March 2024 was performed in PubMed, PsycInfo, PsychArticles, and Scopus Library, using the following string: ‘((“corticosteroids”[Title/Abstract] OR “corticosteroid” [Title/Abstract] OR “steroids”[Title/Abstract] OR “steroid” [Title/Abstract]) AND (“mania” [Title/Abstract] OR “mania episode” [Title/Abstract] OR “manic”[Title/Abstract] OR “manic episode”[Title/Abstract] OR “mania symptoms” [Title/Abstract] OR “mania symptomatology” [Title/Abstract] OR “manic symptoms”[Title/Abstract] OR “manic symptomatology”[Title/Abstract] OR “psychosis” [Title/Abstract] OR “psychotic episode” [Title/Abstract] OR “psychotic symptoms”[Title/Abstract] OR “psychotic symptomatology” [Title/Abstract] OR “bipolar symptoms”[Title/Abstract] OR “bipolar symptomatology”[Title/Abstract] OR “bipolar disorder” [Title/Abstract] OR “psychotic disorder”[Title/Abstract])).”

### 2.2 Study selection

Firstly, duplicates from the different databases were removed. Secondly, a screening of title and abstract was performed and, finally, full text of potentially eligible studies was analyzed. Search, selection, and analysis of the selected studies were performed independently by two reviewers; disagreements were resolved by consensus among these primary raters and a senior investigator. The articles selected and analyzed in this systematic literature review were included based on the following eligibility criteria: (i) original studies (including case reports) investigating clinical populations of at least 18 years of age; (ii) the examined population had a current or past history of corticosteroid therapy at any dosage; (iii) the clinical population under consideration presented manic, psychotic, or both types of side effects; (iv) research article reporting quantitative data. Articles were excluded based on the following exclusion criteria: (i) temporal criterion related to studies published before 2013, considering only the last decade as the temporal range; (ii) studies conducted on the pediatric population; (iii) articles not published in English; (iv) studies that did not mention steroid therapy or any psychiatric side effects, particularly mania and/or psychotic symptoms; (v) articles reporting only qualitative data; (vi) the full-text of the article was not available online even after request to the corresponding author.

### 2.3 Data extraction

Data were independently extracted by both reviewers by means of a precoded form. They included: first author, title, demographics and sample characteristics (i.e., age, gender distribution, disease diagnosis and comorbidities, sample size), type and time of treatment (i.e., corticosteroids and/or steroids), comparison groups, treatment duration (i.e., days, weeks, months), psychiatric side effects (i.e., manic and/or psychotic symptoms).

### 2.4 Data synthesis

Given the heterogeneity of the design of studies (i.e., case reports, case series, and cross-sectional research), as well as the different study population, and the several outcome measures, a meta-analysis was not deemed to be appropriate.

## 3 Results

**FIGURE 1:**
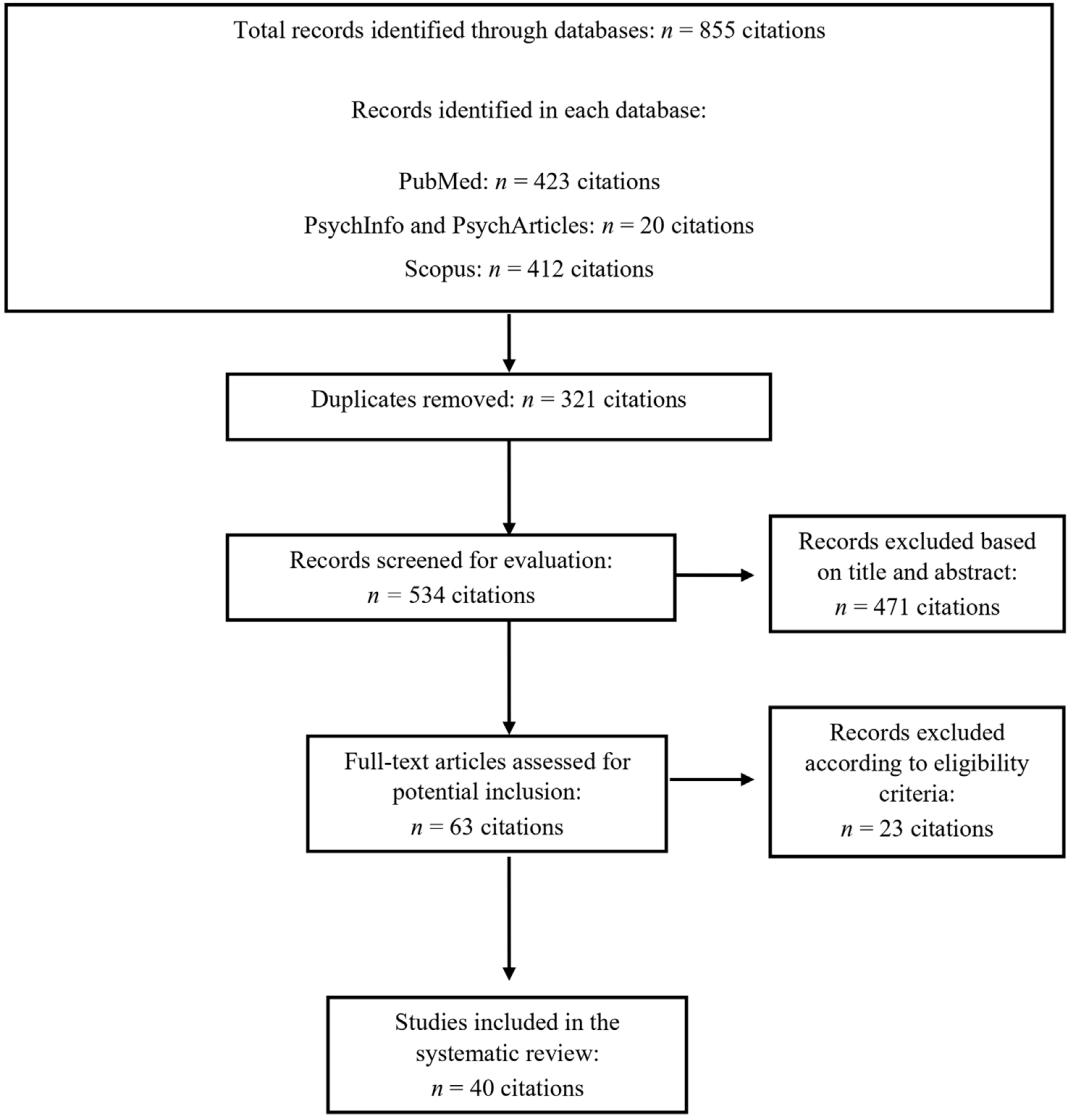
Flowchart of the systematic search.

At the end of the screening process, 40 studies (case reports/case series = 34; quantitative research = 6), were included in the systematic review (see Figure 1 for a detailed description of the study selection process). Detailed results for all studies are reported in Table 1 (case reports/series) and Table 2 (quantitative research). In the majority (i.e., 82.5%) of the reviewed studies, authors only evaluated corticosteroid-induced symptoms of mania and/or psychosis without providing standardized diagnoses based on DSM or ICD diagnostic classification criteria.

**TABLE 1:** Results from case reports and case series.

First author (Year)	Type of paper	Age	Sex	Disease diagnosis	Comorbidities	Sample size	Type of treatment	Dosage of treatment	Treatment and symptoms	Comparison groups	Manic symptoms	Psychotic symptoms	Management of side effects
Alsalem et al. (2022)	Case Report	75 years old	Female	Post-cataract surgery	None reported	1	Prednisolone 1% eye drops	Four times a day	Symptoms appeared 2 days after starting treatment; prednisolone was discontinued after 9 days of use	Not applicable (single case)	Elated mood, pressured speech, flight of ideas, reduced need of sleep, irritability, restlessness, increased motor and verbal activity, talkativeness, irritability	Visual hallucinations	Discontinued prednisolone eye drops; Administered olanzapine 5 mg/day for insomnia and agitation; Rapid improvement within a week, with YMRS score decreasing from 29 to 6; Olanzapine discontinued after 2 months; patient remained stable without psychiatric manifestations
Araujo et al. (2019)	Case Report	69 years old	Male	Large-vessel vasculitis (LVV)	None reported	1	Prednisone	1 mg/kg/day, total of 90 mg/day initially	Symptoms appeared 1 week after starting treatment	Not applicable (single case)	Emotional lability, disorientation, aggressiveness, disinhibition	Hallucinatory activity with a paranoid persecutory theme, cognitive deficit	Weaning corticosteroids and starting new therapy with methotrexate and tocilizumab; Introduction of antipsychotic therapy with olanzapine and quetiapine; Symptoms improved with changes in therapy; continued monitoring and adjustment
Bachu et al. (2023)	Case Series	Case 1:72 years old, Case 2: 23 years old, Case 3: 36 years old	Case 1: Male, Case 2: Male, Case 3: Female	Case 1: Arthritis, Case 2: Ulcerative colitis, Case 3: No specific diagnosis, patients underwent an endoscopy and was started on a regimen of steroids	Not reported	3	Corticosteroids (steroid injection for Case 1, IV methylprednisolone and prednisone for Case 2, unspecified steroids for Case 3)	Case 1: Steroid injection in the elbow (dosage not specified); Case 2: IV methylprednisolone 40 mg thrice daily, then prednisone 40 mg daily; Case 3: Steroid regimen not specified	Symptoms appeared shortly after starting treatment (specific timing varies by case)	Not applicable (case series)	Case 1: Hypervigilance, poor sleep, pressured speech, psychomotor agitation, decreased sense of fatigue. Case 2: Inappropriate laughter. Case 3: Grandiosity, reduced sleep, hyperverbal speech.	Case 1: Paranoia; Case 2: Catatonia, mutism, blunted affect, echopraxia, negativism, thoughts blocking Case 3: Paranoia, disorganized thought processes, delusions.	Case 1: Started on quetiapine, discharged with improved symptoms; Case 2: Treated with benzodiazepines (lorazepam), then antipsychotics (risperidone, olanzapine, clozapine, paliperidone), showed gradual improvement; Case 3: Started on risperidone, then switched to haloperidol, showed significant improvement and discharged on antipsychotic medication
Brykalski et al. (2015)	Case Study	55 years old	Female	Secondary adrenal insufficiency	None reported	1	Hydrocortisone	Initially 400 mg/day intravenously, then reduced to 200 mg/day after 3 days, further reduced after psychotic symptoms appeared	Psychotic symptoms appeared on the fourth day of treatment	Not applicable (single case)	Not specifically mentioned	Hallucinations, delusions, disorganized thinking, agitation, aggressive behavior	Psychiatric consultation and treatment with haloperidol (10 mg/day) and diazepam (up to 15 mg/day); Reduction of hydrocortisone dosage to 10 mg/day; Psychotic symptoms resolved after 3 days of treatment and dosage reduction
El Hayek and El-Khoury (2020)	Case Report	57 years old	Male	Small cell lung carcinoma	Dyslipidemia	1	Prednisone	20 mg daily	Symptoms started 3 weeks after starting treatment and persisted for a few days	Not applicable (single case)	Extreme elation, insomnia, increased energy, disinhibited behaviors, anger outbursts, unwarranted spending, hypersexuality	Grandiose and persecutory delusions	Treatment with risperidone 2 mg daily and bromazepam 1.5 mg twice daily; Discontinuation of psychotropic medications after 11 days as psychiatric symptoms subsided; Subsequent chemotherapy for lung carcinoma with dexamethasone without signs of psychiatric relapse
Fischer and Kim (2019)	Case Report	49 years old	Male	Chronic low back pain and unilateral radicular pain	Anxiety, diabetes, alcoholic cirrhosis status post liver transplant	1	Dexamethasone and lidocaine (epidural steroid injection)	10 mg dexamethasone, 0.5 mL lidocaine	Symptoms started 3 days after treatment and resolved between 7 and 17 days	Not applicable (single case)	Mood swings, insomnia.	Anger, hostility, paranoia, delusions, confusion.	Increased lorazepam dosage temporarily; Prescribed quetiapine as needed for insomnia; Follow-up with primary care physician and health psychologist Symptoms resolved by post-intervention day 17, started on serotonin and norepinephrine reuptake inhibitor (SNRI) and referred to psychiatry
Fujii et al. (2022)	Case Report	66 years old	Female	Crohn’s disease	Chronic kidney disease	1	Prednisolone	10 mg/day	Symptoms started after 6 months of treatment	Not applicable (single case)	Abnormally elevated mood, flight of ideas, decreased need for sleep, increased mental activity, agitation, distractibility	Not specifically mentioned	Treatment with olanzapine (up to 20 mg/day) and sodium valproate (up to 1,000 mg); Reduction and tapering of prednisolone dose; Improvement of manic symptoms with prednisolone tapering; Discontinuation of prednisolone, sodium valproate, and olanzapine after symptom resolution; No relapse of Crohn’s disease or manic episodes for over 4 years
Gable and Depry (2015)	Case Study	58 years old	Male	Idiopathic thrombocytopenic purpura (ITP)	General anxiety disorder	1	Dexamethasone	40 mg bursts every 3 weeks	Symptoms appeared during the third cycle of treatment (approximately 8 weeks after starting), and persisted for nearly 6 months despite cessation of dexamethasone	Not applicable (single case)	Pressured speech, euphoria, hyperactivity, irritability, minimal sleep	Persecutory delusions, auditory hallucinations, disorganized behavior, hyper-religious behaviors (e.g., ‘speaking in tongues’), tangential and circumstantial thought processes.	Initial treatment with low-dose quetiapine, increased to 600 mg at bedtime; Short course of aripiprazole (discontinued due to dystonic reaction); Five days of electroconvulsive therapy (ECT) without improvement; Continuation of high-dose quetiapine for gradual improvement over 5 months
Grover et al. (2021)	Case Series	Case 1: 25 years old, Case 2: 31 years old	Both Male	COVID-19 infection	Case 1: None, Case 2: None	2	Dexamethasone	6 mg/day	Symptoms appeared within a few days of starting treatment	Not applicable (case series)	Case 1: Euphoric to irritable mood, agitation; distractibility. Case 2: Agitation, fearfulness.	Case 1: Grandiose delusions. Case 2: Auditory hallucinations; delusion of persecution	Case 1: Treatment with olanzapine (up to 20 mg/day) and clonazepam (up to 12 mg/day), continuation of dexamethasone due to COVID-19 symptoms; Case 2: Treatment with lorazepam (4 mg/day) and olanzapine (10 mg/day), tapering off dexamethasone after physical condition improved
Hotier et al. (2015)	Case Report	47 years old	Female	Multiple sclerosis	Major depressive episode at age 32, detrusor–external sphincter dyssynergia	1	Methylprednisolone	1 g per day for 3 days	Symptoms started 10 days prior to hospitalization and rapidly improved after methylprednisolone treatment	Not applicable (single case)	Excessive irritability, mood lability, decreased need for sleep, thought and language disturbances (cluttering, flight of ideas)	Paranoid and suspicious thoughts, delusional features	Rapid fluoxetine withdrawal; Introduction of risperidone (2 mg per day); Treatment with intravenous methylprednisolone (1 g per day for 3 days); Significant improvement in manic and delusional features, discharge from the hospital
Ibrahim et al. (2021)	Case Report	36 years old	Male	Hashimoto’s thyroiditis, Hashimoto encephalopathy	None reported	1	Intravenous pulse methylprednisolone, followed by oral prednisolone	1 g intravenous methylprednisolone daily for 5 days, followed by 60 mg oral prednisolone daily with a taper plan of 10 mg monthly	Symptoms improved significantly by the second day of intravenous steroids and resolved completely by the 10th day	Not applicable (single case)	Not specifically mentioned	Auditory hallucinations, delusions of grandeur, paranoia	Intravenous pulse methylprednisolone, followed by oral prednisolone taper; Significant improvement of psychotic features by the second day of intravenous steroids; Complete resolution of symptoms by the 10th day of steroid therapy; Discharged with neurology and endocrinology follow-ups
Ishikawa et al. (2018)	Case Report	70 years old	Male	Tolosa-Hunt syndrome	Hypertension	1	Intravenous methylprednisolone followed by oral prednisolone	1,000 mg/day IV methylprednisolone for 3 days, followed by 60 mg/day oral prednisolone tapered over time	Symptoms of delirium started 5 days after initiating steroid pulse therapy	Not applicable (single case)	Not specifically mentioned	Delirium, clouding of consciousness, speech disturbance, abnormal sleep cycle	Suspension of oral prednisolone treatment (60 mg/day); Resolution of delirium after suspension of prednisolone; Resumption of prednisolone at a lower dose (10 mg/day); No recurrence of delirium or other adverse effects, continued monitoring and gradual tapering
Janes et al. (2019)	Case Report	35 years old	Male	Chronic testicular pain, lumbar spinal stenosis, depression, anxiety, PTSD	Hypertension, hyperlipidemia	1	Dexamethasone injection	5 mg dexamethasone	Symptoms started the day after the injection and lasted approximately 24 h	Not applicable (single case)	Not specifically mentioned	Persecutory delusions, aggressive behavior, paranoia	Emergency department care with physical restraint and emergency detention; Inpatient psychiatric hospitalization recommended; Psychiatric evaluation, supportive care, and monitoring
Jasani et al. (2021)	Case Report	36 years old	Female	Systemic lupus erythematosus (SLE), recent COVID-19 infection	Hypertension	1	Prednisone	30 mg daily (after tapering from a higher dose)	Symptoms appeared on the third day of admission, after resuming prednisone at 30 mg daily	Not applicable (single case)	Elevated mood, decreased need for sleep, distractibility, hyper-religiosity, inappropriate smiling and laughing	Not specifically mentioned	Initiation of valproic acid 500 mg twice a day and clonazepam 1 mg at bedtime as needed; Continuation of prednisone at a reduced dose of 20 mg daily; Gradual improvement and return to baseline mental status within 24 h of starting valproic acid; Plan for gradual tapering of valproic acid in parallel with prednisone taper
Jyothi et al. (2016)	Case Report	30 years old	Male	Addison’s disease, adrenoleukodystrophy (ALD)	None reported	1	Prednisolone	7.5 mg daily for 18 years	Symptoms appeared 14 years after starting prednisolone	Not applicable (single case)	Increased talk, increased self-esteem, over familiar behavior, irritability, overspending, irregularity in job, sleep disturbance	None reported	Initial treatment with sodium valproate (600 mg daily, increased to 1,000 mg daily); Discontinuation of sodium valproate due to ataxia; Treatment with haloperidol (20 mg/day); Significant improvement in manic symptoms, with Young Mania Rating Scale score decreasing from 33 to 14 over 2 weeks
Kassim et al. (2021)	Case Report	37 years old	Male	Sarcoidosis	None reported	1	Intravenous methylprednisolone followed by oral prednisolone	3 days of IV methylprednisolone followed by 60 mg/day oral prednisolone	Symptoms started on the second day of methylprednisolone	Not applicable (single case)	Not specifically mentioned	Auditory hallucinations, persecutory ideations, retrograde amnesia	Treatment with escitalopram, quetiapine, and alprazolam; Continuation of prednisolone despite psychiatric symptoms due to the necessity for sarcoidosis treatment; Significant reduction in psychotic symptoms over time with multidisciplinary management
Kazi and Hoque (2021)	Case Report	55 years old	Female	COVID-19 pneumonia	Type 2 diabetes mellitus, hypertension	1	Intravenous dexamethasone	6 mg IV daily for 7 days	Symptoms appeared 3 days after discharge, following 7 days of treatment	Not applicable (single case)	Agitated behavior, pressured speech, impulsivity, decreased need for sleep, hyperverbal and theatrical behavior	Paranoid grandiose religious delusions	Initial treatment with intravenous diphenhydramine 25 mg, haloperidol 5 mg, and lorazepam 2 mg; Oral haloperidol 5 mg twice daily for psychosis, sodium valproate 500 mg twice daily for mood stabilization, acetylcholine 1 mg twice daily for extrapyramidal symptoms, clonazepam 1 mg twice daily for anxiety and insomnia; Discharge with aripiprazole 10 mg daily and sodium valproate 500 mg twice daily; Follow-up showed no recurrence of symptoms after discontinuation of medications
Khoodoruth and Khan (2020)	Case Report	33 years old	Male	Anabolic Steroids-Induced Delirium	Previously diagnosed with bipolar disorder (2018)	1	Antipsychotics (Haloperidol and Quetiapine)	Haloperidol 7.5 mg/day, Quetiapine 700 mg/day	Treatment administered during hospital stay (25 days) and continued upon discharge	Not applicable (single case report)	Labile affect, irrelevant speech, flight of ideas, increased aggression, hostility, disturbed sleeping patterns.	Persecutory delusions, disorganized behavior, and altered level of consciousness	Managed with antipsychotic medication, which led to significant improvement.
King et al. (2024)	Case Report	Late 50s	Female	Organic bipolar 1-like affective disorder secondary to acquired brain injury, stage 2 breast cancer	None reported	1	Intravenous dexamethasone as part of chemotherapy adjunctive to lumpectomy	16 mg of dexamethasone per day for 3 days	Symptoms appeared several days after the second cycle of chemotherapy	Not applicable (single case)	Elated mood, expansive and labile affect, pressured speech, distractibility, decreased need for sleep, irritability.	Suspected auditory hallucinations, erotomanic delusions, paranoia.	Treatment with Clonazepam 2 mg QDS and Promethazine 25 mg QDS; Continuation of regular mood-stabilizing medications (Lithium and Lamotrigine); Reduction of dexamethasone dosage in collaboration with oncology team; Marked recovery within 7 days, with a plan for adjusted chemotherapy regimen with benzodiazepine cover
Köroğlu and Ayrıbaş (2022)	Case Report	69 years old	Male	COVID-19 pneumonia, mild cognitive impairment	Diabetes mellitus	1	Dexamethasone	8 mg daily	Symptoms appeared after 10 days of dexamethasone treatment	Not applicable (single case)	Euphoria, reduced need for sleep, rapid and increased speech	Grandiose delusions	Initial treatment with haloperidol and quetiapine, switched to olanzapine 15 mg and valproate 1,000 mg; Significant improvement in agitation and psychotic symptoms within a week; Discharged with olanzapine, valproate discontinued due to elevated liver enzymes
Lally et al. (2017)	Case Report	82 years old	Female	Osteoarthritis, moderate dementia (Alzheimer’s type)	Hypertension, hypercholesterolemia, recurrent urinary tract infections, mesenteric infarct (2013), stroke (2009), neck of femur fracture (2008), left Colles’ fracture (2009)	1	Intra-articular corticosteroid injection (DepoMedrone)	80 mg methylprednisolone acetate	Symptoms started within 48 h of injection and lasted 7 days	Not applicable (single case)	Not specifically mentioned	Persecutory delusions, auditory hallucinations, paranoia, agitation, aggressive behavior, personality change	Initial treatment with quetiapine 25 mg twice daily, switched to haloperidol 0.5 mg twice daily due to lack of response; Significant improvement within 4 days of haloperidol, complete resolution within 7 days; Reduction of haloperidol to 0.5 mg once daily for another week, then discontinued
Latvala et al. (2023)	Case Report	70 years old	Female	Giant Cell Arteritis (GCA)	Family history of bipolar disorder and Alzheimer’s disease	1	Corticosteroids	Oral prednisolone 60 mg/day, intravenous methylprednisolone 500 mg/day for 3 days	Initial treatment duration of 8 weeks with gradual dose reduction; full psychiatric symptom recovery in 4–5 months	Not applicable (single case report)	Irritability, elevated mood, motor agitation, and pressured speech.	Delusions, illusions and visual hallucinations.	Valproate dosage was increased to a maximum of 1800 mg/day to control manic and psychotic symptoms. Symptoms gradually improved after 3–4 weeks of continuous treatment, and a full recovery was noted 4–5 months after treatment initiation.
Lesko et al. (2021)	Case Report	Case 1: 77 years old, Case 2: 48 years old, Case 3: 21 years old, Case 4: 65 years old	Case 1: Male, Case 2: Female, Case 3: Female, Case 4: Female	Case 1: Adrenal insufficiency, Hashimoto’s thyroiditis; Case 2: Sheehan’s syndrome; Case 3: Hirsutism; Case 4: Not specified (confusion, disorientation, bizarre behavior)	Case 1: None reported, Case 2: None reported, Case 3: None reported, Case 4: Long-term history of prednisone use	4	Prednisone/Prednisolone	Case 1: 10 mg/day, Case 2: 10 mg/day, Case 3: 5 mg/day, Case 4: 2.5 mg/day	Duration of symptoms varied from immediate onset to several days after starting treatment	Not applicable (case report series)	Case 1: Hyperactivity, agitation, insomnia Case 2: Euphoria, insomnia, restlessness, agitation Case 3: Restless Case 4: Not specifically mentioned	Case 1: Sexual hallucinations, aggression, bizarre speech/behavior Case 2: Visual hallucinations. Case 3: Hallucinations, delusions. Case 4: Persecutory, jealousy, and bizarre delusions, paranoia.	Case 1: Discontinuation of corticosteroids, olanzapine 5 mg BID; Case 2: Discontinuation of prednisolone, lorazepam 4 mg, haloperidol 5 mg; Case 3: Discontinuation of prednisolone; Case 4: Discontinuation of prednisone, olanzapine 2.5 mg BID, haloperidol 2 mg IM daily, benztropine 0.5 mg IM BID, vitamin B12 supplementation
Liu and Wu (2018)	Case Report	29 years old	Female	Major depressive episode, CLIPPERS (Chronic lymphocytic inflammation with pontine perivascular enhancement responsive to steroids)	None reported	1	Intravenous methylprednisolone followed by oral prednisone	500 mg/day IV methylprednisolone for 3 days, tapered to 120 mg/day, followed by prednisone acetate 60 mg/day	Symptoms appeared on the second day following methylprednisolone treatment	Not applicable (single case)	Pressured and rapid speech, inflated self-esteem, overly excitement, decreased need for sleep, increased activities	Not specifically mentioned	Discontinuation of paroxetine; Treatment with quetiapine (200 mg/night) and sodium valproate (1,000 mg/day); Transient depressive episode followed by stabilization
Martinho and Fonseca (2021)	Case Report	55 years old	Male	Posterior uveitis	None reported	1	Oral prednisolone	60 mg/day for 6 weeks	Symptoms started 1 week after abrupt discontinuation of prednisolone	Not applicable (single case)	Severe psychomotor agitation, aggression, pressured speech, decreased need for sleep, extreme irritability, periods of euphoria, disinhibited and impulsive behaviors	Delusions of References, persecution, misidentification	Treatment with risperidone (4 mg/day) and benzodiazepines; Reinstatement and slow reduction of prednisolone to prevent adrenal insufficiency; Significant improvement within 1 week, with the patient recognizing the bizarre nature of his behavior
Misir et al. (2017)	Case Report	25 years old	Male	Systemic lupus erythematosus (SLE), macrophage activation syndrome (MAS)	None reported	1	Intravenous and oral corticosteroids, antipsychotics	16 mg/day oral methylprednisolone	Symptoms appeared after reducing the methylprednisolone dose	Not applicable (single case)	Elevated mood, increased energy, decreased need for sleep, thought acceleration, increased self-confidence, spending sprees	Visual and auditory hallucinations, persecution ideas	Initial treatment with haloperidol, later switched to olanzapine 10 mg/day and valproic acid 1,000 mg/day; Intramuscular zuclopenthixol acetate for agitation and hallucinations; Gradual improvement of symptoms and follow-up for 1 year
Mizutani et al. (2015)	Case Report	35 years old	Male	Pituitary adenoma	None reported	1	Perioperative steroid replacement (hydrocortisone)	200 mg of hydrocortisone initially, tapered to 100 mg, then to 30 mg, and finally 10 mg over 7 days	Symptoms appeared on day 4 post-surgery	Not applicable (single case)	Elated mood, agitation	Grandiose delusions, persecution mania, aggressive behavior	Treatment with risperidone (initially 0.5 mg/day, increased to 2 mg/day); Symptoms gradually improved and disappeared by postoperative day 14; No recurrent psychiatric symptoms for 10 months post-episode
Moss et al. (2014)	Case Report	89 years old	Male	Chronic obstructive pulmonary disease (COPD)	Atrial fibrillation, hypertension, posttraumatic stress disorder (PTSD), depression, chronic kidney disease	1	Budesonide/formoterol combination inhaler	160 mg budesonide/4.5 mcg formoterol, 2 puffs twice daily	Symptoms appeared within 1 week of starting the combination inhaler	Not applicable (single case)	Not specifically mentioned	Confusion, hallucinations (visual and auditory)	Discontinuation of the combination inhaler led to resolution of confusion and hallucinations; Rechallenge during hospitalization confirmed the association; Management included monitoring by the medical team and discontinuation of the offending inhaler
Nagano et al. (2019)	Case Report	80 years old	Female	Rheumatoid arthritis, organising pneumonia	Vasospastic angina	1	Isoniazid preventive therapy, prednisolone	5 months Isoniazid 200 mg/day, prednisolone (initially 45 mg, then reduced to 17.5 mg)	Mania appeared after 5 months of isoniazid therapy	Not applicable (single case)	Agitation, disturbed sleep, inability to rest	Delusion, violence, incoherent and disorganized speech,	Discontinuation of isoniazid on day 3 of hospitalization led to resolution of psychotic symptoms within 1 week; Prednisolone dose was increased to 20 mg on day 8 without aggravating psychotic symptoms; Commenced rifampicin for latent tuberculosis, patient discharged
Nishimura et al. (2014)	Case Series	Mean age 36.2 years (range 19–62 years)	All female	Systemic lupus erythematosus (SLE)	Not specified in detail for each patient	135	Corticosteroids (prednisolone)	Mean dosage 0.98 mg/kg/day (range 0.24–1.39 mg/kg/day)	8 weeks follow-up, psychiatric events occurred within a mean of 12 days (range 2–28 days)	Not applicable (case series)	9 cases with manic features, 2 cases with mixed features	1 case with psychotic disorder	14 psychiatric events suitable for the definition of corticosteroid-induced psychiatric disorders (CIPDs), resolved with corticosteroid dosage reduction; 2 cases of CNS-SLE, required augmentation of immunosuppressive therapy; EEG abnormalities and elevated cerebrospinal fluid (CSF) markers (IL-6, IFN-α) were indicative of CNS-SLE in some cases
Perez et al. (2021)	Case Report	39 years old	Male	COVID-19 pneumonia	Depression, anxiety, posttraumatic stress disorder (PTSD), childhood asthma, obstructive sleep apnea, smoker	1	Dexamethasone	6 mg IV daily during hospitalization for 9 days, continued 6 mg oral daily for 2 days post-discharge	Symptoms appeared 2 days after discharge	Not applicable (single case)	Grandiose thoughts, elevated mood, pressured speech, increased irritability, hyperactivity, insomnia	Religious delusions, hallucinations, paranoia.	Initial treatment with lorazepam and olanzapine; Discharged on olanzapine 5 mg daily, failed to obtain medication; Subsequent re-hospitalization for acute mania and psychosis, treated with olanzapine 10 mg daily and valproate 1,000 mg daily; Gradual improvement over hospitalization, continued follow-up, and medication adjustment; Complete resolution of symptoms at 3-month follow-up
Roxanas (2018)	Case Report	Case 1: 67 years old, Case 2: 72 years old	Case 1: Male, Case 2: Female	Case 1: Toxoplasmosis vitreoretinitis, Case 2: Chest infection	Case 1: Hypertension, benign prostatic hypertrophy, sclerosing cholangitis; Case 2: Breast cancer (mastectomy), asthma, atrial fibrillation	2	Prednisone	Case 1: Prednisone 75 mg daily, tapered over 6 weeks; Case 2: Prednisone 50 mg daily, tapered over 2 weeks	Symptoms appeared during tapering and persisted for several months after stopping corticosteroids	Not applicable (single case)	Case 1: Increased appetite, restlessness, increased libido, insomnia, irritability, labile mood, pressured speech. Case 2: Inappropriate laughter, insomnia, aggression, disorganized behavior, pressured speech	Case 1: Delusions Case 2: Not specifically mentioned	Case 1: Sodium valproate (500 mg twice daily, increased to 1,000 mg twice daily) and quetiapine (200 mg at night); symptoms controlled within 5 months; Case 2: Sodium valproate (500 mg twice daily); symptoms controlled within a few days, medication stopped after 3 months; Both patients were well at 1-year follow-up
Sevim et al. (2015)	Case Report	25 years old	Female	Multiple sclerosis (MS), bipolar disorder	None reported	1	High-dose methylprednisolone	1,000 mg/day intravenous methylprednisolone for 5 days	Symptoms improved within 3 days of steroid treatment	Not applicable (single case)	Elevated mood, inappropriate jokes, amplified gestures, mild increase in libido, irritability, increased speech, anger	Not specifically mentioned	Initial recommendation for quetiapine (2 × 50 mg) which the patient refused; Manic symptoms unexpectedly improved without antipsychotics after 3 days of steroid treatment; Complete improvement of hemiparesis after 5 days
Ward and George (2016)	Case Report	62 years old	Male	Rheumatoid arthritis	Hypertension, ischemic heart disease, long-standing mild anxiety and depression	1	Methylprednisolone injection	One-off dose of 80 mg methylprednisolone	Symptoms appeared 1 week after the steroid injection	Not applicable (single case)	Agitation, irritability, pressured speech, disjointed speech, inappropriate remarks	Persecutory delusions	Initial manual restraint and intramuscular lorazepam for physical aggression; aripiprazole was started but refused by the patient; Depot injection of zuclopenthixol and valproic acid were administered, leading to improvement; Discharged on valproic acid and zuclopenthixol, with continued follow-up

**TABLE 2:** Results from quantitative research.

First author (Year)	Age	Sex	Disease diagnosis	Comorbidities	Sample size	Type of treatment	Dosage of treatment	Treatment and symptoms	Comparison groups	Manic symptoms	Psychotic symptoms	Management of side effects
Alturaymi et al. (2023)	Mean age 47.06 ± 17.093 years	1,082 males (34.5%), 2056 females (65.5%)	Various inflammatory and autoimmune diseases	Not specified	3,138	Oral corticosteroids	Mean dose 7.05 ± 6.627 mg	Mean duration of oral corticosteroid use was 93.01 ± 54.91 days	Not applicable (descriptive study without control groups)	Not specifically mentioned	2 patients (0.06%)	Monitoring patients for signs of mental health problems; Adjusting treatment as needed; Educating patients about the potential risks associated with corticosteroids
Lotan et al. (2016)	Mean age 50.3 years (SD 15.6 years)	63.3% female	Various neurological disorders (optic neuritis, myelitis, multiple sclerosis, neuromyelitis optica, chronic inflammatory demyelinating polyneuropathy, pain syndromes)	Not specified	49	High-dose intravenous corticosteroids	1,000 mg of intravenous methylprednisolone daily for 5 days (inflammatory conditions), 500 mg of intravenous methylprednisolone daily for 3 days (pain syndromes)	Evaluations before, immediately after, and 1 month following treatment	Patients with inflammatory neurological disorders vs patients with pain syndromes	Transient increase in Young Mania Rating Scale scores immediately after treatment, returned to baseline after 1 month	No significant increase in Brief Psychiatric Rating Scale scores, overall reduction observed	Monitoring using Beck Depression Inventory, Geriatric Depression Scale, Young Mania Rating Scale, and Brief Psychiatric Rating Scale; Statistically significant reduction in depressive symptoms 1 month after treatment; No clinically significant affective or psychotic side effects observed during the study period
Migita et al. (2013)	Mean age 59.1 years (SD 16.9 years)	59.3% female	Various autoimmune diseases including rheumatic diseases, systemic lupus erythematosus, mixed connective tissue disease, polymyositis, dermatomyositis, vasculitis, Behçet disease, systemic scleroderma, adult-onset Still disease, Sjögren syndrome, rheumatoid arthritis, autoimmune bullous diseases, anaphylactoid purpura, multiple sclerosis, myasthenia gravis, chronic inflammatory demyelinating polyneuropathy, ulcerative colitis, autoimmune hepatitis, autoimmune pancreatitis, primary biliary cirrhosis, idiopathic interstitial pneumonia, collagen vascular disease, rapidly progressive glomerulonephritis, chronic glomerulonephritis, nephrotic syndrome	Various, including diabetes, cardiovascular diseases, interstitial lung disease	604	Glucocorticoids (GCs)	Mean GC dose for the first month was 50.4 mg/day prednisolone	Patients were followed for a mean of 1.9 years	High-dose (≥30 mg/day) vs low-dose (<30 mg/day) glucocorticoid treatment	Not specifically mentioned	23 cases of steroid psychosis observed during the follow-up period	Management included hospitalization for serious adverse events, treatment with antibiotics for infections, and monitoring of psychiatric symptoms
Morrow et al. (2015)	Mean age 39.7 years (SD 10.3 years)	67% female, 33% male	Multiple sclerosis (MS)	37.5% with current or past depression, 11.4% with substance abuse history	88	High-dose corticosteroids (HDC)	1,000 mg/day intravenous methylprednisolone or 1,250 mg/day oral prednisone for 3–5 days	Symptoms monitored before treatment, 3 days after treatment, and 1 month after treatment	Not applicable (single group)	Increased hypomanic symptoms in 38.2% of subjects, including increased energy, rapid speech, and decreased need for sleep	Not specifically mentioned	Symptoms were monitored using the Mood Disorders Questionnaire (MDQ) and Beck Depression Inventory-Fast Screen (BDIFS); Symptoms were transient and generally returned to baseline 1 month after treatment; Personal history of depression and low quality of life were predictors of increased hypomanic symptoms
Shimizu et al. (2016)	Mean age 37.4 years (SLE patients), 52.4 years (other systemic autoimmune diseases)	83.6% female (SLE patients), 63.6% female (other systemic autoimmune diseases)	Systemic lupus erythematosus (SLE), other systemic autoimmune diseases (polymyositis/dermatomyositis, adult-onset Still’s disease, vasculitis syndrome)	Antiphospholipid syndrome (APS), past history of mental disorder	146 with SLE, 162 with other systemic autoimmune diseases, 43 with *de novo* NPSLE	High-dose corticosteroids (≥40 mg/day of prednisolone), immunosuppressive therapies	Prednisolone ≥40 mg/day, methylprednisolone pulse therapy, intravenous cyclophosphamide	NP manifestations occurred within a median of 12 days (SLE patients), 20 days (other systemic autoimmune diseases)	SLE patients vs other systemic autoimmune diseases, PSNP-SLE vs *de novo* NPSLE	Mood disorder more frequent in PSNP-SLE than in *de novo* NPSLE	Psychosis observed in both PSNP-SLE and *de novo* NPSLE	Intensified immunosuppressive treatments (methylprednisolone pulse, intravenous cyclophosphamide, other immunosuppressants); Psychotropic drugs (e.g., risperidone, quetiapine, paroxetine, lorazepam, sodium valproate); Better prognosis for PSNP-SLE compared to *de novo* NPSLE, with higher event-free survival rates
Yagi et al. (2021)	Mean age 62.5 years (SD 17.8 years)	61.3% female	Various conditions requiring corticosteroid therapy	25.8% with past history of psychiatric disorders	93	Oral prednisolone	High-dose (≥0.5 mg/kg/day) and low-dose (<0.5 mg/kg/day) groups	Psychiatric conditions occurred during hospitalization	High-dose vs low-dose oral prednisolone groups	Not specifically mentioned	4 cases with steroid-induced psychosis (high-dose: 3, low-dose: 1); 25 cases with steroid-induced delirium (high-dose: 5, low-dose: 20)	Psychiatric referrals and treatments by consultation-liaison psychiatry team; No significant differences in most psychiatric conditions between high-dose and low-dose groups, except higher incidence of delirium in low-dose group

### 3.1 Characteristics of the sample

The reviewed studies involved adult patients over 18 years (Moss et al., 2014; Sevim et al., 2015; Lally et al., 2017; Misir et al., 2017; Nagano et al., 2019; Grover et al., 2021; Lesko et al., 2021; 2021; Bachu et al., 2023). Female percentage was higher in both the 34 case reports/series and in the six original studies. Regarding the quantitative studies, the total sample consisted of 4,118 patients, with a mean female percentage of 77.2%. The most common medical conditions treated with corticosteroids were systemic lupus erythematosus (SLE) (Migita et al., 2013; Nishimura et al., 2014; Shimizu et al., 2016; Misir et al., 2017; Jasani et al., 2021), rheumatoid arthritis (RA) (Migita et al., 2013; Ward and George, 2016; Nagano et al., 2019), multiple sclerosis (MS) (Hotier et al., 2015; Sevim et al., 2015; Lotan et al., 2016), as well as respiratory conditions (Moss et al., 2014; Misir et al., 2017; Kazi and Hoque, 2021; Perez et al., 2021), such as chronic obstructive pulmonary disease (COPD) and pneumonia, which were more prevalent in females.

Comorbidities were common among the study populations, with 9 case reports revealing the presence of psychiatric conditions prior to corticosteroid therapy, such as depression, anxiety, and post-traumatic stress disorder (PTSD) (Moss et al., 2014; Hotier et al., 2015; Fischer and Kim, 2019; Janes et al., 2019; Perez et al., 2021; Bachu et al., 2023; Latvala et al., 2023). Such pre-existing conditions were often exacerbated by corticosteroid use, contributing to the complexity of managing symptoms of mania and/or psychosis in this clinical population (Sevim et al., 2015; Ward and George, 2016).

### 3.2 Characteristics of the studies

#### 3.2.1 Case reports and case series

The 34 case reports and case series provided detailed accounts of individual patients who developed symptoms of mania and/or psychosis following corticosteroid therapy, based on systemic and/or topic administration. The corticosteroids most frequently associated with these psychiatric side effects included prednisolone (Nishimura et al., 2014; Araujo et al., 2019; Nagano et al., 2019; Ibrahim et al., 2021; Kassim et al., 2021; Lesko et al., 2021; Martinho and Fonseca, 2021; Alsalem et al., 2022; Fujii et al., 2022; Latvala et al., 2023) and methylprednisolone (Hotier et al., 2015; Sevim et al., 2015; Ward and George, 2016; Lally et al., 2017; Misir et al., 2017; Ishikawa et al., 2018; Liu and Wu, 2018; Ibrahim et al., 2021; Bachu et al., 2023). Corticosteroids dosages ranged from low-dose therapy (e.g., <0.5 mg/kg/day of prednisolone) to high-dose therapy (e.g., ≥1 mg/kg/day), with manic and/or psychotic symptoms often emerging within days to weeks of treatment initiation (Nishimura et al., 2014; Mizutani et al., 2015; Ward and George, 2016).

As shown in Table 1, symptoms of mania and psychosis often coexist. In 22 of the 34 reviewed case reports, patients under corticosteroids were found to exhibit symptoms of both mania and psychosis. In two-thirds of the clinical cases, patients showed symptoms of mania with mood-congruent psychotic features, whereas in only a few of them symptoms of mania were associated with mood-incongruent psychotic features. In four of the case reports, patients only presented with manic symptoms, while in eight of the clinical cases patients only showed psychotic symptoms. Symptoms of both mania and psychosis appear within a short time frame after the initiation of corticosteroid therapy, typically ranging from days to weeks. For example, in the study by Mizutani et al. (2015), a 35-year-old male patient developed acute psychotic symptoms in addition to elated mood just 4 days after receiving high-dose hydrocortisone during surgery for a pituitary adenoma. The patient exhibited elated mood, irritation, grandiose delusions (i.e., insisting he was a rich person), persecution mania (i.e., insisting someone had embedded microchip in his brain), anxiety, aggressive behavior, and agitation, thus showing signs and symptoms of steroid-induced mania with psychosis (Mizutani et al., 2015). Similarly, Ward and George (2016) reported a case where a 62-year-old male with rheumatoid arthritis presented with a combination of corticosteroid-induced psychiatric symptoms of mania and psychosis. The patient exhibited severe psychotic symptoms, including persecutory delusions (i.e., believing he was a target of racism), agitation and irritability, approximately 1 week after an 80 mg injection of methylprednisolone (Ward and George, 2016). In another case report, Nagano et al. (2019) observed that in an 80-year-old patient with rheumatoid arthritis and organizing pneumonia, symptoms of mania (e.g., abnormal behavior characterized by agitation, and violence) and psychotic symptoms (e.g., delusions, disorganized and incoherent speech) appeared after 5 months of high-dose corticosteroid therapy. These findings suggest that while these corticosteroid-induced psychiatric symptoms of mania and psychosis can often coexist and manifest rapidly, the exact timing of onset varies depending on factors such as the corticosteroid dosage and the underlying health condition of the patient (Mizutani et al., 2015; Ward and George, 2016; Nagano et al., 2019).

The most common corticosteroid-induced psychiatric side effects reported across the case reports included: (i) mania, characterized by elevated mood, emotional lability, grandiosity, hyperactivity, and decreased need for sleep (Nishimura et al., 2014; Gable and Depry, 2015; Hotier et al., 2015; Mizutani et al., 2015; Sevim et al., 2015; Jyothi et al., 2016; Ward and George, 2016; Misir et al., 2017; Liu and Wu, 2018; Roxanas, 2018; Araujo et al., 2019; Nagano et al., 2019; El Hayek and El-Khoury, 2020; Khoodoruth and Khan, 2020; Grover et al., 2021; Jasani et al., 2021; Kazi and Hoque, 2021; Lesko et al., 2021; Martinho and Fonseca, 2021; Perez et al., 2021; Alsalem et al., 2022; Fujii et al., 2022; Köroğlu and Ayrıbaş, 2022; Latvala et al., 2023; King et al., 2024); (ii) psychosis, manifesting as delusions, hallucinations, and disorganized thinking (Moss et al., 2014; Nishimura et al., 2014; Brykalski et al., 2015; Gable and Depry, 2015; Hotier et al., 2015; Mizutani et al., 2015; Ward and George, 2016; Lally et al., 2017; Misir et al., 2017; Ishikawa et al., 2018; Roxanas, 2018; Araujo et al., 2019; Fischer and Kim, 2019; Janes et al., 2019; Nagano et al., 2019; El Hayek and El-Khoury, 2020; Khoodoruth and Khan, 2020; Grover et al., 2021; Ibrahim et al., 2021; Kassim et al., 2021; Kazi and Hoque, 2021; Lesko et al., 2021; Martinho and Fonseca, 2021; Perez et al., 2021; Alsalem et al., 2022; Köroğlu and Ayrıbaş, Başar, 2022; Bachu et al., 2023; Latvala et al., 2023; King et al., 2024); (iii) depression. While less common than mania and psychosis, some patients experienced depressive episodes characterized by low mood, anhedonia, and suicidal ideation. Kassim et al. (2021) reported the case of a 37-year-old male with sarcoidosis who, on his second day of treatment with methylprednisolone, abruptly developed partial retrograde amnesia followed by 3 months of depressive symptoms (i.e., low mood with loss of interest, prominent hopelessness and worthlessness) with panic attacks, which remitted with an antidepressant and a benzodiazepine. However, 2 months later, the patient started experiencing persecutory ideations and a mix of second and third-person auditory hallucinations which were not mood congruent (Kassim et al., 2021).

In several case reports, psychiatric symptoms of mania and psychosis persisted even after the discontinuation of corticosteroid therapy, necessitating long-term treatment with antipsychotics, mood stabilizers, or other medications. Roxanas (2018) illustrated this phenomenon with two cases of elderly patients who developed persistent mania following corticosteroid cessation. One patient, a 67-year-old male, continued to experience manic symptoms (i.e., increased anger, appetite, libido, grandiose ideas, restlessness, and decreased need for sleep) for 3 months after discontinuing corticosteroids, necessitating long-term treatment with mood stabilizers and antipsychotics (Roxanas, 2018). The second patient was a 72-year-old woman with manic symptoms (i.e., inappropriate laugh, insomnia, aggressive and disorganized behavior) that persisted for 4 months after stopping prednisone (Roxanas, 2018). Similarly, Gable and Depry (2015) presented the case of a 58-year-old man where the coexisting corticosteroid-induced symptoms of mania and psychosis (e.g., pressured speech, tangential, confused, and circumstantial thought) persisted despite cessation of corticosteroid therapy, necessitating prolonged psychiatric care including antipsychotics and ECT to achieve symptom resolution.

Moreover, a few reports observed that when corticosteroids were reintroduced after being discontinued, the psychiatric symptoms reappeared. For example, Jasani et al. (2021) described a patient who experienced corticosteroid-induced mania (i.e., elevated mood, decreased need for sleep, distractibility, hyper-religiosity, and inappropriate smiling and laughing) after previously tolerating higher doses without psychiatric symptoms. The symptoms emerged when corticosteroids were reintroduced. Similarly, Moss et al. (2014) reported a case where a male patient with chronic obstructive pulmonary disease experienced hallucinations while on corticosteroids. After his symptoms resolved following the cessation of corticosteroid therapy, confusion and hallucinations reappeared when the corticosteroids were administered again during hospital admission.

Furthermore, the persistence of psychiatric symptoms after corticosteroid therapy varied considerably across the case reports and case series. In several instances, psychiatric symptoms resolved relatively quickly once the corticosteroid dosage was reduced or the therapy was discontinued. For example, in the case report by Brykalski et al. (2015), a 55-year-old female patient with secondary adrenal insufficiency developed acute psychotic symptoms (i.e., auditory and visual hallucinations, delusions of thought insertion, religious and bizarre delusions, and disorganized thinking) after being treated with high-dose corticosteroids. The patient was also agitated, verbally and physically aggressive toward other persons, thus exhibiting symptoms of mania (Brykalski et al., 2015). Her symptoms of delusions and agitation resolved within 3 days after tapering off the corticosteroids, and she required only short-term antipsychotic treatment. Another example is provided by Fujii et al. (2022), who described a 66-year-old female with Crohn’s disease who developed corticosteroid-induced mania after long-term treatment with low-dose (i.e., 10 mg/day) prednisolone. Her manic symptoms, which included abnormally elevated mood, flight of ideas, decreased need for sleep, severe agitation and distractibility, were resistant to initial treatment with olanzapine and sodium valproate but resolved after the corticosteroid dose was tapered. The patient did not experience further psychiatric symptoms of mania following the discontinuation of prednisolone (Fujii et al., 2022).

#### 3.2.2 Quantitative research

The six research studies provided broader insights into the prevalence and predictors of mania and/or psychosis in patients taking corticosteroids. As shown in Table 2, in two of the six original studies, patients under corticosteroids were found to exhibit symptoms of both mania and psychosis. In one study, patients only displayed symptoms of mania (elated mood), while in 3 studies patients only presented a diagnosis of psychosis.

The corticosteroids most frequently associated with psychiatric symptoms of mania and/or psychosis included prednisolone (Migita et al., 2013; Shimizu et al., 2016; Yagi et al., 2021; Alturaymi et al., 2023) and methylprednisolone (Morrow et al., 2015; Lotan et al., 2016), with corticosteroids dosages ranging from low-dose therapy (e.g., ≤0.5 mg/day) to high-dose therapy (e.g., >10 mg/day).

Across the studies, the incidence of psychiatric side effects varied but was generally reported between 10% and 30%. Sleep disturbance emerged as the most commonly reported psychiatric side effect. For example, in Yagi et al. (2021), according to DSM-5, 72% of patients experienced insomnia following corticosteroid therapy, while 4.3% was diagnosed with steroid-induced psychosis, and 15.1% presented with corticosteroid-induced mania. Shimizu et al. (2016) reported that approximately 25% of patients treated with corticosteroids developed mood disturbances, and other 25% of the sample was diagnosed with steroid-induced psychosis. In another research study, Alturaymi et al. (2023) found that from 5% to 18% of patients developed new-onset psychiatric disorders with only two patients out of 3,138 steroid users who developed psychosis.

Methylprednisolone was particularly associated with mania and psychosis in higher doses. For example, Lotan et al. (2016) found that patients receiving methylprednisolone for autoimmune conditions developed significant psychiatric symptoms, including mania and psychosis. Migita et al. (2013) observed similar results in patients with autoimmune disease who were treated with methylprednisolone. In this cohort study (Migita et al., 2013), the authors showed that 3.8% presented with only psychosis.

### 3.3 Corticosteroids dosage

The dosage of corticosteroids varied widely across the studies, with both high-dose and low-dose regimens being associated with symptoms of mania and/or psychosis (Gable and Depry, 2015; Ward and George, 2016; Lesko et al., 2021). The specific dosage often played a crucial role in determining the type and severity of psychiatric symptoms reported by patients.

Moderate to high-dose corticosteroid therapy was frequently associated with more severe symptoms of mania and/or psychosis. For instance, Gable and Depry (2015) reported that the patient receiving high doses of corticosteroids (greater than 40 mg of prednisone or its equivalent) developed significant psychiatric symptoms of mania and psychosis, including hyperactivity, pressured and unintelligible speech, hallucinations and delusional thinking, which persisted for nearly 6 months despite cessation of corticosteroids. Similarly, Ward and George (2016) reported a case of severe psychosis associated with symptoms of mania (e.g., agitation, irritability, and pressured speech) following high-dose methylprednisolone therapy in a patient with rheumatoid arthritis. The patient developed significant psychiatric symptoms, including persecutory delusions, shortly after receiving an 80 mg injection of methylprednisolone, requiring intensive psychiatric care (Ward and George, 2016).

Lower-dose regimens were also implicated in corticosteroid-induced symptoms of mania and/or psychosis. For instance, Lesko et al. (2021) reported a case series of four patients (age range: 21–77 years old) all treated with prednisone: one patient at 2.5 mg/day; one patient at 5 mg/day; and two patients at 10 mg/day, for few days before the interruption of treatment due to psychiatric side effects, that included agitation, bizarre speech, unusual and self-harm behavior, hyperactivity, delusions, hallucinations. Similarly, Fujii et al. (2022) detailed a case where a 66-year-old female with Crohn’s disease developed severe elevated mood, flight of ideas, agitation, distractibility, and insomnia after being treated with prednisone at a dosage of 10 mg/day for 6 months. While symptoms described in both studies (Lesko et al., 2021; Fujii et al., 2022) were less severe compared to those induced by higher doses of corticosteroid, they still required intervention with benzodiazepines, such as lorazepam, to provide symptomatic relief.

In quantitative studies, the variability in corticosteroids dosage and its associations with psychiatric symptoms of mania and/or psychosis were also noted, however with inconsistent results. For example, in the study by Yagi et al. (2021) patients were administered oral prednisolone and divided into two groups, namely high-dose and low-dose group. There were no statistical differences between the two groups for several psychiatric symptoms, only delirium was found to be statistically more associated with low-dose of corticosteroids (N = 20) rather than high-dose (N = 5).

### 3.4 Pharmacological management of corticosteroid-induced mania and/or psychosis

The pharmacological management of corticosteroid-induced psychiatric symptoms of mania and/or psychosis varied widely across the studies, reflecting the heterogeneity of symptoms and the different patient populations. The most common pharmacological interventions included the use of antipsychotics, mood stabilizers, and benzodiazepines, each tailored to address specific psychiatric symptoms.

Antipsychotics were frequently employed to manage severe symptoms of mania and psychosis, particularly in cases where these symptoms posed a significant risk to the patient’s safety or functioning. For example, Köroğlu and Ayrıbaş (2022) reported the use of olanzapine associated with a mood stabilizer in a COVID-19 patient presenting with corticosteroid-induced symptoms of mania and psychosis (e.g., grandiose delusions, elevated mood, psychomotor agitation, reduced need for sleep, rapid and increased speech). Olanzapine (15 mg) and valproate (1,000 mg) were effective in controlling the patient’s symptoms of mania and psychosis, which gradually declined in a week (Köroğlu and Ayrıbaş, 2022).

Mood stabilizers, such as valproic acid and lithium, were commonly employed to address mood disturbances during corticosteroid therapy. Kazi and Hoque (2021) reported a case of a 55-year-old patient who developed symptoms of mania and psychosis (i.e., psychomotor agitation, pressured speech, and paranoid and grandiose delusions) after steroid administration. The patient was effectively treated with sodium valproate (i.e., 500 mg twice daily), a mood stabilizer, in combination with other medications (i.e., haloperidol, 5 mg twice daily for psychosis, acetylcholine, 1 mg twice daily to prevent extrapyramidal symptoms, and clonazepam, 1 mg twice daily for anxiety and insomnia) (Kazi and Hoque, 2021). The treatment regimen successfully stabilized the patient’s mood and managed the manic and psychotic symptoms (Kazi and Hoque, 2021). Additionally, King et al. (2024) described the successful use of lithium in a female patient with stage-2 breast cancer who developed corticosteroid-induced symptoms of mania and psychosis (e.g., auditory hallucinations, elated mood, irritability, paranoid ideas, pressured speech). Lithium, in combination with another mood stabilizer (i.e., lamotrigine) and with sedative medications (i.e., clonazepam and promethazine), helped to control her mood swings and prevented further episodes of mania and psychosis, allowing for the safe continuation of corticosteroid therapy as needed given her clinical condition (King et al., 2024).

Benzodiazepines were often used to manage symptoms such as agitation, anxiety, and insomnia, which frequently accompanied corticosteroid-induced psychiatric symptoms. For instance, Bachu et al. (2023) described the case of a 23-year-old patient with steroid-induced signs and symptoms of catatonia and psychosis, where lorazepam (2 mg twice daily), a benzodiazepine, was used effectively to manage catatonic symptoms. The patient was initially treated with lorazepam to control agitation and stabilize the condition before transitioning to other antipsychotic medications, thus supporting the clinical utility of benzodiazepines in the acute management phase (Bachu et al., 2023).

In addition to pharmacological interventions, gradual tapering of corticosteroids was consistently emphasized across the studies as a reasonable strategy to minimize the risk of psychiatric symptoms of mania and/or psychosis. For instance, in Araujo et al. (2019), a 69-year-old male with large-vessel vasculitis developed symptoms of psychosis (e.g., hallucinatory activity with a paranoid persecutory theme) due to high-dose corticosteroid therapy. The patient’s symptoms improved when the corticosteroids were gradually tapered, and antipsychotic medications were introduced alongside steroid-sparing agents like methotrexate and tocilizumab. Similarly, Ishikawa et al. (2018) reported a case of a 70-year-old male with Tolosa-Hunt syndrome who developed delirium during corticosteroid therapy. The patient’s delirium resolved after temporarily halting corticosteroids, followed by a cautious resumption at a lower dose, demonstrating the effectiveness of a gradual reduction in dosage to manage psychiatric symptoms (Ishikawa et al., 2018).

### 3.5 Risk factors and moderators

The reviewed studies highlighted several sociodemographic factors associated with an increased risk of corticosteroid-induced psychiatric symptoms of mania and/or psychosis.

Age emerged as a significant moderator, with elderly patients being particularly vulnerable to corticosteroid-induced symptoms of mania and/or psychosis. For example, Shimizu et al. (2016) found that older adults with systemic autoimmune diseases were more likely to develop post-steroid neuropsychiatric (PSNP) symptoms of psychosis, given a number of age-related changes in the brain, including reduced neuroplasticity, and the presence of comorbid conditions such as dementia. Similarly, in a case report by Moss et al. (2014), an 89-year-old male developed hallucinations and severe delirium, shortly after initiation of corticosteroid therapy, underscoring how advanced age and underlying cognitive impairments can significantly increase individual susceptibility to corticosteroid-induced psychiatric symptoms of psychosis. Roxanas (2018) reported the cases of elderly patients who exhibited prolonged manic symptoms following corticosteroid cessation, further indicating that age-related factors play a crucial role also in the persistence of corticosteroid-induced psychiatric symptoms of mania.

Studies have also focused on gender as a factor that may increase the individual susceptibility to corticosteroid-induced psychiatric symptoms of mania and/or psychosis. Alturaymi et al. (2023) observed that women were at higher risk for corticosteroid-induced psychiatric symptoms of psychosis.

Pre-existing psychiatric conditions seem to consistently emerge as a significant risk factor. Patients with a history of mental health disorders, such as depression or anxiety, appear to have an increased risk of developing mania and/or psychosis during corticosteroid therapy. For instance, Ward and George (2016) described a 62-year-old male with a history of mild anxiety and depression who developed psychosis after corticosteroid therapy, requiring intensive psychiatric intervention. Liu and Wu (2018) presented a case of a 29-year-old woman with a history of major depressive disorder who developed corticosteroid-induced symptoms of mania (i.e., pressured and rapid speech, inflated self-esteem, decreased need for sleep and increased activities) following corticosteroid therapy (i.e., methylprednisolone).

Longitudinally, the persistence and recurrence of psychiatric symptoms of mania and/or psychosis following corticosteroid therapy were well-documented across several studies, underscoring the importance of extended monitoring and follow-up care for affected patients. For instance, Lesko et al. (2021) described cases where patients developed psychiatric symptoms of psychosis, after corticosteroids were reintroduced. It was found that even at lower doses, the reintroduction of corticosteroids led to the emergence of new psychiatric symptoms, including agitation, hallucinations, and mood disturbances. This highlights the potential for corticosteroids to induce psychiatric side effects upon re-exposure, regardless of the dosage. Similarly, Nishimura et al. (2014) reported that psychiatric symptoms of mania and/or psychosis can manifest within a few days of corticosteroid administration and persist over time, lasting for a minimum of 14 days to 240. This study also showed that these symptoms can recur even after the initial resolution, particularly during subsequent corticosteroid courses, thereby further underscoring the importance of extended monitoring and follow-up care to manage long-term psychiatric outcomes effectively (Nishimura et al., 2014).

Finally, the role of cumulative corticosteroid exposure seems to be another critical factor in understanding the development of chronic psychiatric conditions. Alturaymi et al. (2023) showed that patients undergoing prolonged corticosteroid therapy, specifically those treated for more than 28 days, were significantly more susceptible to developing mental health disorders, including corticosteroid-induced psychiatric symptoms of psychosis. This study found that even after tapering off corticosteroids, psychiatric symptoms often continued or recurred, suggesting a lasting impact of extended corticosteroid exposure (Alturaymi et al., 2023). These findings suggest the importance of considering the duration of corticosteroid treatment as a risk factor for chronic psychiatric conditions and the need for long-term monitoring and follow-up care to manage corticosteroid-induced psychiatric side effects (Alturaymi et al., 2023).

### 3.6 Can corticosteroids improve manic and/or psychotic symptoms?

In the context of corticosteroid therapy for multiple sclerosis (MS), two case reports (Hotier et al., 2015; Sevim et al., 2015) presented rare exceptions where such treatment has led to the improvement of corticosteroid-induced psychiatric symptoms of mania and/or psychosis. Both cases involved young female patients with long-standing histories of MS. The patient in Sevim et al. (2015) study was 25 years old, while the patient in the Hotier et al. (2015) study was 47 years old. The fact that both patients were women could suggest that demographic factors might influence both the presentation of MS and the psychiatric effects. Furthermore, both patients had experienced multiple relapses and remissions over several years, highlighting the chronic nature of their disease and the repeated exposure to corticosteroid treatments. In both cases (Hotier et al., 2015; Sevim et al., 2015), the patients’ manic symptoms, which might typically be exacerbated by corticosteroids, instead improved significantly during the course of treatment. In Sevim et al. (2015) a 25-year-old woman with a 7-year history of MS and co-occurring bipolar disorder refused antipsychotic treatment but displayed marked improvement in manic symptoms by the third day of corticosteroid therapy. Manic symptoms (i.e., elevated mood, mild increased in libido, irritability, and pressured speech) of the patient, instead of being exacerbated by the ongoing treatment with 1,000 mg/day of methylprednisolone, significantly improved (Sevim et al., 2015). Similarly, in the case presented by Hotier et al. (2015) corticosteroid treatment (i.e., intravenous methylprednisolone, 1 g per day, during 3 days) led to the alleviation of corticosteroid-induced psychiatric symptoms of mania and psychosis (i.e., paranoid and suspicious thoughts associated with excessive irritability in the presence of marked mood lability, a decreased need for sleep, and thought and language disturbances with flight of ideas) in an MS patient, a finding that challenges the conventional understanding of corticosteroid-induced psychiatric side effects.

Despite these similarities, there are, however, important differences between the two cases that should be considered (Hotier et al., 2015; Sevim et al., 2015). First, the patients’ history, particularly the pre-existing psychiatric condition, should be taken into consideration. In Sevim et al. (2015) the patient had a documented history of bipolar disorder alongside MS, which played a central role in the clinical interpretation of her symptoms and in the treatment planning. In this case, the authors (Sevim et al., 2015) specifically addressed the interaction between bipolar disorder and MS, noting the heightened risk of psychiatric symptoms such as mania in patients with both conditions. In contrast, the case reported by Hotier et al. (2015) had no a pre-existing psychiatric condition. Instead, the patient’s manic symptoms were directly associated with the course of MS, suggesting a different etiology or perhaps a more acute neuropsychiatric manifestation of MS itself (Hotier et al., 2015). Finally, these cases highlight the complexity of corticosteroid effects in neuropsychiatric contexts, suggesting that, in certain patients, corticosteroids may have a stabilizing effect on psychiatric symptoms of mania and psychosis, particularly within the MS population.

## 4 Discussion

The findings of this systematic review show that in 11.8% of clinical cases and 1 (out of six studies) quantitative research patients under corticosteroids presented with manic symptoms only. In 23.5% of clinical cases and in three quantitative studies, patients had psychotic symptoms only. The findings of this systematic review also indicate that manic and psychotic symptoms often coexist in patients taking corticosteroids (Brykalski et al., 2015; Gable and Depry, 2015; Mizutani et al., 2015; Ward and George, 2016; Nagano et al., 2019; Lesko et al., 2021; Martinho and Fonseca, 2021; Yagi et al., 2021). Indeed, in 64.70% of clinical cases, and in 33.3% of research studies, patients under corticosteroids presented with symptoms of both mania and psychosis. Of note, one of the studies included in the present systematic review showed that corticosteroid-induced symptoms of psychosis can emerge in about 30%–40% of cases during a manic episode (Gable and Depry, 2015). These findings are consistent with a previous study, in which it was found that 73% of manic patients treated with steroids had psychotic symptoms (Lewis and Smith, 1983). Prevalence rates reported in our systematic review are also in line with another study (Wada et al., 2001), where the authors showed that, of patients with a corticosteroid-induced mood disorder, 40% had mania with psychotic features.

In contrast with the usual yet questionable practice of primarily directing clinical attention to “steroid psychosis” (Hall et al., 1979; Dubovsky et al., 2012), which represents an inaccurate and misleading term including not only psychotic but also affective symptoms, the current review has the potential to pave the ground to a better use of the psychopathological terminology, supporting the need to establish a better understanding of the etiology, treatment, and outcome of neuropsychiatric complications of corticosteroids (Dubovsky et al., 2012). In addition, the present review supports the frequent association between corticosteroid-induced symptoms of mania and psychosis, as documented in other studies (Mullen and Romans-Clarkson, 1993; Türktaş et al., 1997). However, there remains a need to clarify the direction and nature of this relationship, for example to establish which symptoms appear first and their interaction and progression over time. The present review is the first to apply clinimetric methods in order to enhance a longitudinal understanding of the relation between corticosteroid-induced symptoms of mania and psychosis. More specifically, macro-analysis, a clinimetric method that has been developed for organizing clinical data (Emmelkamp, 2004), may help clarify the functional relationship between co-occurring symptoms of mania and psychosis that may be part of underlying clinical conditions in patients receiving corticosteroids. This clinimetric procedure has the potential to enable clinicians and researchers to determine which symptoms came first, placing corticosteroid-induced symptoms of mania and psychosis into a hierarchy and thus giving priority to the treatment of the most severe symptoms.

The use of macro-analysis requires the application of the staging method (Fava, 2024), a clinimetric procedure that may provide an essential perspective regarding the course and longitudinal development of corticosteroid-induced symptoms of mania and psychosis, differentiating the prodromal, acute, and residual phases of these clinical manifestations.

To better understand the development and overtime interaction between corticosteroid-induced symptoms of mania and psychosis, Rome and Braceland (1952) were the first authors to propose a staging model of corticosteroid-induced psychiatric side effects. Their model included the following four stages: the prodromal phase or stage 1 characterized by mild euphoria, reduced subjective perception of fatigue, improved concentration, and elevated mood; stage 2, where the patient is effusive, expansive, volatile, active, hypomanic, and also exhibits flight of ideas, impaired judgment, insomnia, and increased appetite; stage 3, in which the patient may experience a wide range of symptoms, including anxiety, phobia, rumination, obsessional preoccupations, hypomania, and depression; and finally stage 4, the phase in which the patient exhibits symptoms of psychosis, mainly delusions and hallucinations that, according to Rome and Braceland (1952), tended to subside spontaneously within a few weeks after the discontinuation of the corticosteroid therapy.

Over the years, researchers have made limited use of this staging model (Rome and Braceland, 1952) given the relatively low number of studies applying this classification to the evaluation of the course and progression of corticosteroid-induced symptoms of mania and psychosis. The application of this staging model to clinical practice and research may provide unique clinical information regarding longitudinal trajectories of symptoms of mania and psychosis in patients taking corticosteroids. It should be noted, however, that such a staging model (Rome and Braceland, 1952) needs to be tested and eventually updated/revised, particularly to verify its current applicability to current corticosteroid therapies.

Other methodological flaws affecting most of the reviewed studies include the following: (1) the limited use of diagnostic criteria leading to a considerable inter-clinician variability in the assessment of corticosteroid-induced symptoms of mania and psychosis; thus, simple reference to corticosteroid-induced mania and/or psychosis without a clear distinction between symptoms of mania/psychosis and manic/psychotic disorders is no longer acceptable in future studies; this implies the urgent need of specific standardized diagnostic criteria that should involve the use of clinician-rated and self-reported questionnaires to ensure not only a comprehensive assessment of these clinical phenomena but also an adequate differential diagnosis between primary and corticosteroid-induced mania with mood-congruent/incongruent psychotic features; (2) the majority of the reviewed studies provided cross-sectional or short-term follow-up data, thus limiting the ability to draw firm conclusions regarding the long-term effects of corticosteroid-induced symptoms of mania and/or psychosis; (3) most of the reviewed studies were case reports, which often lack control groups; this may limit the generalizability of findings of the present review, thus implying the need for further research based on more rigorous study designs (e.g., randomized placebo-controlled trials). Therefore, caution should be paid in the interpretation of the findings of the present review, particularly in psychiatry where there is the urgent need to replicate the results based on larger clinical samples.

Clinical pharmacopsychology, a scientific approach that is concerned with the application of clinimetric methods to the assessment of psychotropic effects of medications (Fava et al., 2017), may provide valuable conceptual and methodological guidelines to address current research gaps in the evaluation of the presentation and short- and long-term course of corticosteroid-induced mania and/or psychosis. Indeed, the application of this emerging approach in clinical practice and research has the potential to improve the prediction of individual vulnerabilities induced by treatment, allowing an early detection of corticosteroid-induced mania and/or psychosis.

Regarding the most significant risk factors that may increase the individual susceptibility to corticosteroid-induced symptoms of mania and/or psychosis, five main indications emerge from the present systematic review.

First, corticosteroid-induced symptoms of mania and psychosis are associated with prolonged therapy, thus patients receiving chronic corticosteroid therapy are at higher risk of developing these severe psychiatric symptoms (Dubovsky et al., 2012; Nasereddin et al., 2024; Tavares et al., 2024). However, clinicians should be aware that corticosteroid-induced mania and/or psychosis may also develop after short-term exposure with symptoms that can emerge within the first week of treatment in up to 90% of patients (Naber, 1996; Dubovsky et al., 2012; Gable and Depry, 2015; Oray et al., 2016; Sofía-Avendaño-Lopez et al., 2024). Of note, a study by Robinson et al. (2000), in which, 36 hours after a single dose of an intra-articular injection of steroids, a 75-year-old woman with hip osteoarthritis and no history of psychiatric disorder or dementia developed paranoid delusions, visual and auditory hallucinations (Robinson et al., 2000).

Second, patients who receive high-dose of corticosteroids (i.e., usually defined as greater than 40 mg of prednisone or its equivalent) are at higher risk of developing symptoms of mania and/or psychosis (Lewis and Smith, 1983; Gable and Depry, 2015; Nasereddin et al., 2024; Sofía-Avendaño-Lopez et al., 2024). However, extreme caution should be paid given that even small doses of corticosteroids (e.g., typically defined as lower than 7.5 mg of prednisone or its equivalent) have been reported to induce symptoms of mania and/or psychosis (Thibaut, 2019; Lesko et al., 2021; Nasereddin et al., 2024).

Third, pre-existing psychiatric conditions were found to be associated with increased risk for corticosteroid-induced symptoms of mania and/or psychosis (Fishman et al., 1996; Morrow et al., 2015; Sofía-Avendaño-Lopez et al., 2024). However, there is evidence in the literature (Patten and Neutel, 2000; Dubovsky et al., 2012; Gable and Depry, 2015) suggesting that corticosteroid-induced symptoms of mania and/or psychosis can even occur in patients who had no personal or familial psychiatric history. A case report (Martinho and Fonseca, 2021) where a 55-year-old man, with no personal or familial psychiatric history, in 1 week after discontinuation of corticosteroid therapy developed severe symptoms of mania and psychosis, characterized by psychomotor agitation, pressured speech, decreased need for sleep, extreme irritability and euphoria, delusions of reference and persecution. The emergence of manic and psychotic symptoms after steroid withdrawal can thus also occur.

Fourth, age is an additional risk factor, with elderly patients who seem to be more susceptible to experiencing corticosteroid-induced symptoms of mania and/or psychosis (Moss et al., 2014; Shimizu et al., 2016; Roxanas, 2018). Of note, however, the findings of a comprehensive review of 79 published case reports and 14 unpublished clinical cases, in which the authors (Lewis and Smith, 1983) found that the mean age was 39.6 years for patients who developed corticosteroid-induced symptoms of mania and/or psychosis. Thus, this issue deserves further investigation.

Fifth, females appear to be at higher risk for developing corticosteroid-induced symptoms of mania and/or psychosis (Chen et al., 2021; Alturaymi et al., 2023). However, in their recent review, De Bock and Sienaert (2024) noted that there is a lack of consensus regarding female gender as a risk factor for corticosteroid-induced symptoms of mania and/or psychosis and recommended further exploration on this subject.

Future studies, especially meta-analyses, providing a quantitative estimate of the overall likelihood of these adverse events, are needed.

Another controversial issue deserving further investigation has to do with tapering. There are no Food and Drug Administration (FDA)-approved guidelines for the treatment of steroid-induced psychiatric symptoms but, given the risk of psychotic mania after sudden or abrupt discontinuation of high-dose corticosteroids (Martinho and Fonseca, 2021), slow tapering has been suggested to manage and prevent these side effects (Lu, 2021).

It should, however, be noted that the idea that by gradual reduction clinicians can decrease the risk or even avoid psychiatric side effects is, in the case of corticosteroids, not always supported by the literature (Oray et al., 2016; Lesko et al., 2021; Fujii et al., 2022; Tavares et al., 2024). Indeed, many studies revealed that psychosis can also occur during slow tapering of corticosteroids (Oray et al., 2016; Tavares et al., 2024).

As a concluding remark regarding the discussion of risk factors, it is clinically worth noting that, while the duration of treatment, dosage, pre-existing psychiatric conditions, age, gender, and tapering may increase risk, these clinical elements do not determine which patients will experience symptoms of mania and/or psychosis during a given course of corticosteroid therapy. In practical terms, this implies that all patients should be considered to have the potential of experiencing and manifesting corticosteroid-induced symptoms of mania and/or psychosis, and they should therefore be constantly monitored during therapy with corticosteroids and also assessed at the end of treatment to quantify the risk of developing these psychiatric side effects.

Some further indications, which may be clinically useful to improve the detection of corticosteroid-induced symptoms of mania and/or psychosis, emerge from the present systematic review. Corticosteroid-induced mania and/or psychosis are not only highly prevalent but also persistent symptoms (Migita et al., 2013; Lotan et al., 2016; Araujo et al., 2019; Martinho and Fonseca, 2021; Alsalem et al., 2022; Köroğlu and Ayrıbaş, Başar, 2022) that do not necessarily disappear with discontinuation of the drugs (Gable and Depry, 2015; Roxanas, 2018; Jasani et al., 2021). In other words, corticosteroid-induced mania and/or psychosis symptomatology can begin during the treatment, can persist despite the cessation of the corticosteroid therapy (Nishimura et al., 2014; Gable and Depry, 2015; Lesko et al., 2021; Alturaymi et al., 2023) and, furthermore, can initiate following its discontinuation (Martinho and Fonseca, 2021), thus representing a particularly insidious form of behavioral toxicity.

The clinical evaluation of behavioral toxicity (DiMascio and Shader, 1968; Fava et al., 2016), a concept that encompasses adverse events that may ensue from drug administration or cessation and/or persist long even after the discontinuation of the treatment, has received relatively little clinical attention. In patients taking corticosteroids, the attention of prescribers was mainly on baseline risk of poor outcomes rather than on the longitudinal assessment of individual vulnerability to corticosteroid-induced symptoms of mania and psychosis.

This appraisal may not emerge unless corticosteroid-induced symptoms of mania and/or psychosis are prospectively monitored and adequately assessed based on follow-up evaluations over the course of treatment and the end of therapy. Such repeated evaluations should involve the use of highly sensitive clinimetric indices. The clinimetric property of sensitivity, a concept that was introduced for the first time in 1972 by Robert Kellner, refers to the ability of a rating scale to reflect psychological states (whether symptoms or well-being) and their changes with treatment (Carrozzino et al., 2021), including subtle changes that may ensue with long-term therapy. In their recent systematic review and meta-analysis, Koning et al. (2024) advocated the use of patient-reported outcome measures (PROMs) to assess synthetic glucocorticoid-related neuropsychiatric adverse effects. Such a clinical recommendation should be extended to the evaluation of corticosteroid-induced symptoms of mania and/or psychosis in relation to the different types of corticosteroids.

Over the years, a number of PROMs have been developed and can therefore be used in clinical practice and research, particularly those that were found to entail the clinimetric property of sensitivity, to improve the longitudinal detection of corticosteroid-induced symptoms of mania and/or psychosis (Lindström et al., 2001; Wisniewski et al., 2006).

It should be noted, however, that rating scales, including the most sensitive ones, do not replace clinical judgment and reasoning (Fava et al., 2012) of experienced clinicians, representing essential components for a comprehensive assessment of the seriousness, longitudinal characteristics and impact of corticosteroid-induced symptoms of mania and/or psychosis.

## 5 Conclusion

Given the wide variation in the clinical manifestations and experiences of corticosteroid-induced symptoms of mania and/or psychosis, as well as their unpredictable course and multifactorial etiology, a generic diagnostic rubric that applies to the “average” patient in all clinical situations for the different types of corticosteroids independently from the dosage of the drug and the duration of the treatment is no longer acceptable.

Clinicians prescribing corticosteroids must be aware that each case may be different and take advantage of clinimetric methods, which may pave the ground for a substantial improvement in the early detection and evaluation of severity of corticosteroid-induced symptoms of mania and/or psychosis.

The methodological recommendations, introduced for the first time in this systematic review, have the great potential to allow clinical investigators and practitioners (1) to adequately assess and monitor the likelihood of responsiveness to a certain type and dose of corticosteroid in the individual patient, (2) to early recognize the prodromal manifestations of corticosteroid-induced symptoms of mania and/or psychosis, (3) to assess problems of functional capacity and the impact of these symptoms on psychological well-being and quality of life of patients, and (4) to quantify and thus minimize the risk that patients under corticosteroids may have to develop symptoms of mania and/or psychosis.
